# PABPN1 as a pan-cancer biomarker: prognostic significance and association with tumor immune microenvironment

**DOI:** 10.3389/fimmu.2025.1553527

**Published:** 2025-06-18

**Authors:** Hong-Xing Li, Xiao-Ling Ma, Wang-bin Ma, Tian-Yu Jia, Xiao-Hong Sun, Xiao-Xia He, Li-li Zhang, Ya-Ming Xi

**Affiliations:** ^1^ The First School of Clinical Medicine, Lanzhou University, Lanzhou, China; ^2^ Clinical Medical Research Center for Reproductive Diseases of Gansu Province, Lanzhou, China; ^3^ Department of Hematology, The First Hospital of Lanzhou University, Lanzhou, China; ^4^ Department of General Surgery, Renmin Hospital of Wuhan University, Wuhan, China

**Keywords:** PABPN1, biomarker, prognostic, immune microenvironment, cancer

## Abstract

**Objective:**

This study aimed to investigate the role of poly(A) binding protein nuclear 1 (PABPN1) as a potential pan-cancer biomarker for prognosis and immunotherapy.

**Methods:**

The original datasets were acquired from TCGA and GEO databases. PABPN1 expression analysis was conducted utilizing the Oncomine, TIMER, GEPIA, and BioGPS databases. Prognostic implications of PABPN1 were assessed through GEPIA, Kaplan-Meier plotter, and the PrognoScan database. Correlations between PABPN1 expression and immune checkpoints (ICP), tumor mutational burden (TMB), microsatellite instability (MSI), and neoantigens in human cancers were examined using the SangerBox database. Additionally, the association between PABPN1 and marker genes of tumor-infiltrated immune cells in urogenital cancers was confirmed. Differential expression of PABPN1 in urogenital cancers with distinct clinical characteristics was assessed using the UALCAN database. Finally, experiments of T24, 5637, HLF and MCF-7 cells were performed to verify the above results.

**Results:**

The expression of PABPN1 tended to be higher in human cancers compared to paired normal tissues. Its expression levels showed strong associations with TMB, MSI, and neoantigens. Additionally, significant correlations existed between PABPN1 expression and tumor immune-infiltrated cells (TILs) in many human cancers, with marker genes of TILs showing significant relationships with PABPN1 expression, particularly in urogenital cancers. The coexpression networks of PABPN1 were predominantly involved in the regulation of immune response, antigen processing, and presentation. After down expression of PABPN1, mRNA expression levels of MRPS15 and GPx (Glutathione peroxidase) decreased significantly in T24, 5637 HLF and MCF-7 cells.

**Conclusion:**

PABPN1 was expected to be an important role element in cancer research, serving as a potential prognostic and immunological pan-cancer biomarker.

## Introduction

The maturation process of eukaryotic precursor mRNAs (pre-mRNAs) involved cleavage and polyadenylation (C/P) at their 3’ end (1). In eukaryotes, a majority of genes had multiple polyadenylation signals (PASs), giving rise to alternative cleavage and polyadenylation (APA). The selection of APA resulted in the generation of diverse transcript isoforms characterized by variable 3’ untranslated region (3’UTR) lengths and sequence compositions. The 3’UTR played a crucial role in influencing mRNA stability, translation efficiency, transportation, and cellular localization. APA standed out as a significant post-transcriptional regulatory mechanism in eukaryotes. Several trans-acting factors involved in APA regulation have been identified to date, including symplekin, RNA polymerase II (RNAP II), poly(A) polymerase (PAP), and nuclear poly(A)-binding protein 1 (PABPN1). Their intricate interactions contributed to the fine-tuning of APA, thereby exerting control over the diversity of transcript isoforms and the subsequent functional consequences on gene expression in eukaryotic cells (2, 3).

The poly(A) tails served a crucial role in the post-transcriptional regulation of gene expression and were consistently associated with PABPNs. Initially, PAP gradually synthesized 10 to 15 adenylate residues at the 3’ end of the precursor mRNA. Subsequently, PABPN1 bound to the short poly(A) sequence, providing an anchor for PAP, which then rapidly synthesized additional adenylate residues. This process resulted in the formation of a poly(A) tail typically consisting of about 200 adenosine residues (4). Additionally, PABPN1 played a role in promoting the utilization of distal PAS. *In vitro* studies indicated that PABPN1 inhibited cleavage at proximal mRNA PAS. The removal or inhibition of PABPN1 leaded to a global shortening of 3’UTRs (5). Moreover, PABPN1 modulated mRNA export transport through interacting with Aly/REF export factor (ALYREF) and binding to the 3′UTR of mRNA (6, 7).

Earlier investigations have demonstrated a notable global shortening of 3’UTRs in cancer cell lines in comparison to tumor samples. This observation suggested that PABPN1 might be one of the key regulators influencing APA profiles across many cancer types (8). For example, Zhou et al. (9) identified a correlation between PABPN1 expression and overall survival (OS) in patients with gastric cancer. In non-small cell lung cancer, the dysregulation of PABPN1 may contribute to tumor aggressiveness by potentially releasing cancer cells from microRNA-mediated gene regulation (10). In prostate cancer, a novel risk model comprising five genes, namely poly(rC) binding protein 1 (PCBP1), PABPN1, protein tyrosine phosphatase receptor type F (PTPRF), differentiation antagonizing non-protein-coding RNA (DANCR), and MYC, was established for predicting progression-free survival (PFS). This model was developed using a publicly available TCGA dataset, achieving an area under the curve (AUC) ranging from 0.64 to 0.78 (11). In glioblastoma cells, the knockdown of Bcl2l2-Pabpn1 by targeting its fusion junction resulted in reduced expression, leading to the suppression of cell proliferation, migration, and invasion *in vitro* (12). In cervical cancer, the overexpression of PABPN1 reversed the inhibited cancer development and radio-resistance induced by the miR-1323 inhibitor (13). The knockout of Pabpn1 resulted in a swift and substantial depletion of hematopoietic stem and progenitor cells (HSPCs) as well as myeloid cells, ultimately causing severe blood diseases (14). Moreover, in a study by Zhao et al. (15), circ-PABPN1 was identified as a potential novel molecular mechanism through which propofol represses colorectal cancer development. These findings collectively suggested that PABPN1 may be expected to be a valuable pancancer biomarker with potential prognostic and therapeutic implications.

However, there has been limited systematic analysis of the role of PABPN1 in prognosis and immunology across many human cancers. This study aimed to comprehensively investigate the involvement of PABPN1 in both prognosis and immunology in human cancers. We explored the potential associations between PABPN1 expression and immune subtypes, molecular subtypes in different cancer types, promising immune biomarkers, and tumor-infiltrating cells (TILs) in the tumor microenvironment (TME). Furthermore, we extended our analysis to urogenital cancers to validate the findings in human cancers. The primary objective of this study was to uncover the potential of PABPN1 in anticancer immunotherapy for human cancers, thereby providing insights into a novel antitumor strategy.

## Methods

### Data and software availability

Data were sourced from The Cancer Genome Atlas (TCGA) (https://cancergenome.nih.gov/) and Gene Expression Omnibus (GEO) (https://www.ncbi.nlm.nih.gov/geo/) databases. Specific details about human cancers and individual sample sizes can be found in **Supplementary Table S1**. Descriptions of the online tools employed were provided below.

### Analysis of PABPN1 expression in human cancers

PABPN1 expression in human cancers and paired normal tissue was compared using the TIMER 2.0 database (http://timer.comp-genomics.org/) and the GEPIA database (http://gepia2.cancer-pku.cn/#analysis) (16–18). Expression profiles of PABPN1 in various cancer and paired normal cell lines were analyzed using the BioGPS database (http://biogps.org) (19).

### Analysis of the prognostic value of PABPN1 in human cancers

The prognostic value of PABPN1 expression in human cancers was explored using the GEPIA database (http://gepia2.cancer-pku.cn/#analysis) and the Kaplan-Meier Plotter database (http://kmplot.com/analysis/) (18, 20). The GEPIA database, an online platform that utilizes tumor and normal tissue data from TCGA, was employed to examine the correlation between PABPN1 expression and OS and disease-free survival (DFS) across 33 cancer types. Group classification was based on the median PABPN1 expression as a cutoff in GEPIA database. Additionally, the Kaplan-Meier Plotter database, which automatically determined optimal cutoff values, was used to identify associations between PABPN1 expression and OS and relapse-free survival (RFS) in 21 cancer types. The analysis included the calculation of hazard ratios (HRs) with corresponding 95% confidence intervals (CIs) and log-rank P-values.

### Analysis of PABPN1 expression in immune and molecular subtypes of human cancers

The TISIDB database (http://cis.hku.hk/TISIDB/index.php) served as an online integrated repository portal that aggregates extensive human cancer datasets derived from the TCGA database (21). Through the TISIDB database, we investigated the correlations between PABPN1 expression and immune or molecular subtypes across various cancer types. Statistically significant differences were defined as those with a P-value < 0.05.

### Microsatellite instability, neoantigen, and ESTIMATE of the TME in human cancers

The association between PABPN1 expression and aspects of the TME was investigated using the Sangerbox website (http://sangerbox.com/tool.html) and bioinformatics website (http://bioinformatics.com.cn). Sangerbox online platform utilized TCGA data for subsequent analysis (22). Tumor mutational burden (TMB), MSI, and neoantigens were established biomarkers of the TME (23, 24). The ESTIMATE algorithm (Estimation of Stromal and Immune cells in Malignant Tumor tissues using Expression data), designed for predicting tumor purity in the TME, included stromal score (indicating the presence of stroma in tumor tissue), immune score (reflecting the infiltration of immune cells in tumor tissue), and estimate score (inferred tumor purity) (25). The SangerBox website was utilized to explore the correlations between PABPN1 expression and these TME biomarkers. Statistical significance was defined at a P-value < 0.05.

### The correlation between PABPN1 expression and immune infiltration cells and their marker genes

Initially, we investigated the relationship between PABPN1 expression and six distinct immune cell types (B cells, CD4+ T cells, CD8+ T cells, neutrophils, macrophages, and dendritic cells) within the TME across 31 human cancers using the SangerBox website. Subsequently, we extended our analysis to explore the correlation between PABPN1 expression and a broader spectrum of ten immune cell types within the TME. This extended examination was conducted specifically in six urogenital cancers. The ten immune cell types encompassed B cells lineage, CD8+ T cells, cytotoxic lymphocytes, endothelial cells, fibroblasts, monocytic cells, myeloid dendritic cells, neutrophils, natural killer cells, and T cells. To validate the findings, we conducted a reanalysis of the correlation between PABPN1 expression and TILs using both the TIMER and GEPIA databases. The TIMER database, comprising a comprehensive dataset of 10,897 samples across 32 cancer types from TCGA, was specifically designed to assess the extent of tumor-associated immune cell infiltration within TME (26). Following that, we proceeded to confirm the correlation between PABPN1 expression and 24 TILs in six urogenital cancers through the GEPIA database. Subsequently, the TIMER database was utilized to investigate the relationship between PABPN1 expression and marker genes associated with key immune cell types (27–29). Statistical significance was attributed to differences with a P-value < 0.05.

### PABPN1 genomic alterations in six urogenital cancers

The cBio Cancer Genomics Portal (c-BioPortal) (http://cbioportal.org) aggregated a multidimensional cancer genomics dataset (30). In our study, we utilized the c-BioPortal database to investigate genomic alterations of PABPN1 in six urogenital cancers.

### PABPN1 expression in different clinical subgroups of six urogenital cancers

The UALCAN database (http://ualcan.path.uab.edu) compiled RNA-seq and clinical data from 31 cancer types obtained from TCGA (31), providing a valuable platform for the analysis of gene expression in both tumor and normal tissues. In our study, we utilized this database to investigate the relationship between individual gene expression and clinicopathological features across various human cancers.

### Analysis of PABPN1 coexpression networks

The LinkedOmics database (http://www.linkedomics.org/login.php) served as a visual platform and was utilized to explore the gene expression profile in this study (32). In our analysis, LinkedOmics was employed to identify coexpression genes of PABPN1 using Pearson’s correlation coefficient. The results were visually presented through heat maps and volcano plots. Additionally, we delved into the Gene Ontology biological process (GO_BP) and Kyoto Encyclopedia of Genes and Genomes (KEGG) pathways of PABPN1 and its coexpression genes through gene set enrichment analysis (GSEA).

### Cell culture and MTT assay

T24, 5637, HLF and MCF-7 cells were maintained in DMEM medium containing 10% FBS at 37°C, and 5% CO_2_. Transient transfection was performed with Lipofectamine 2000 reagent (Invitrogen) according to the manufacturer’s instructions. After incubated with MTT reagent (50 μg/ml) for 4 h, treatment medium was removed and the observance was measured at 570 nm using microplate reader from Versa Max Molecular Device (Sunnyvale, CA). This study was approved by the Ethics Committee of the First Hospital of Lanzhou University (LDYYLL2022–130).

### RNA isolation and RT-PCR

Bladder cancer tissues and para-carcinoma tissues were collected. Total RNA was isolated from tissues and cells using Trizol reagent (Invitrogen) as suggested by the manufacturer. cDNA was synthesized using Quantscript RT Kit (Tiangen). RT-PCR was performed with Power-Up SYBR Green Master Mix (Thermo Fisher). The CT (Threshold cycle) values were analyzed by Thermo Scientific Piko Real software (Thermo Fisher). The siRNA and PCR primers used in the experiments were shown in **Supplementary Table S2**.

### WB assay

Total protein was extracted from cells or tissues using RIPA lysis buffer (CWBIO, Jiangsu, China). WB assays were performed according to the guidelines provided by Abcam (https://www.abcam.cn/protocols/general-western-blot-protocol-2) utilizing a Bio-Rad gel analysis system (Bio-Rad, Hercules, CA, USA).WB bands were quantified using the ImageJ software(NIH, Bethesda, MD, USA).

### Colony formation assay

The cells were grown in 6-well dishes with a cell density of 1000 cells per well for a period of 14 days. After the 14-day duration, the cells were immobilized with 4% paraformaldehyde and subjected to crystal violet staining (Beyotime, Shanghai, China). The ability of the cell colony to form was subsequently evaluated based on the presence of colonies.

### Statistical analysis

GraphPad Prism 8.3.0 was utilized for the purpose of conducting the data analysis. The values are presented as the mean ± standard deviation. The differences between two groups were assessed using unpaired or paired Student’s t-test. The examination of the relationships between two variables utilized Pearson’s correlation coefficient. The statistical significance was established by considering *P < 0.05, **P < 0.01, ***P < 0.001, ****P < 0.0001, or ns (indicating no significance).

## Results

### Significant differential expression of PABPN1 was observed between tumors and normal tissues in many human cancers

**Figure 1:**
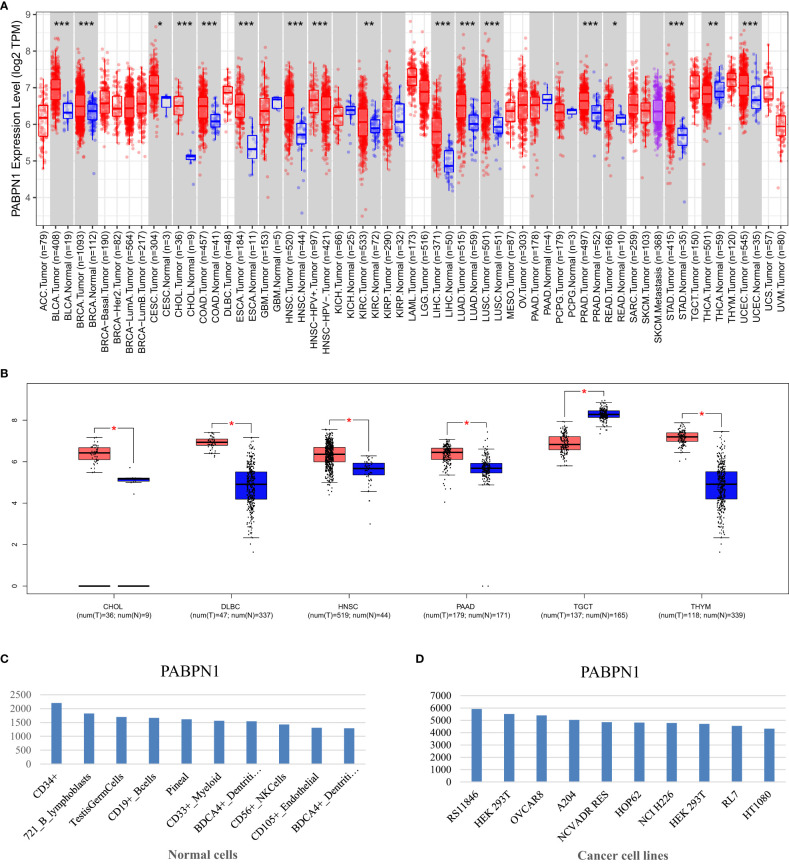
PABPN1 expression levels in human cancers. **(A)** Analysis of PABPN1 expression levels in cancer types from the TCGA database using the TIMER tool. **(B)** Comparison of PABPN1 expression in several cancers and their paired normal tissues using the GEPIA database. **(C)** Assessment of PABPN1 expression in different cancer cell lines utilizing the BioGPS database. **(D)** Examination of PABPN1 expression in normal tissues based on the BioGPS database. *P < 0.05, **P < 0.01, ***P < 0.001, the same below.

The TIMER database analysis revealed significantly higher expression levels of PABPN1 in multiple human cancers, including BLCA (bladder urothelial carcinoma), BRCA (breast invasive carcinoma), CESC (cervical cancer), CHOL(cholangiocarcinoma), COAD (colon adenocarcinoma), ESCA(esophageal carcinoma), HNSC (head and neck cancer), KIRC (kidney clear cell carcinoma), KIRP (kidney renal papillary carcinoma), LIHC (liver hepatocellular carcinoma), LUAD (lung adenocarcinoma), LUSC (lung squamous cell carcinoma), PRAD (prostate adenocarcinoma), READ (rectal cancer), STAD (stomach adenocarcinoma), THCA (thyroid cancer) and UCEC (uterine corpus endometrial carcinoma), compared to adjacent normal tissues (**Figure 1A**). And the results from the GEPIA database analysis, focusing on cancers without paired normal tissues in the TIMER database, demonstrated significantly elevated PABPN1 mRNA expression in CHOL (cholangiocellular carcinoma), DLBC (large B-cell lymphoma), PAAD (pancreatic adenocarcinoma), and THYM (thymoma) except TGCT (testicular cancer) (**Figure 1B**). The BioGPS database analysis was conducted to examine the expression of PABPN1 across various cancer cell lines and normal tissues. The findings revealed a consistently high expression level of PABPN1 in almost all cancer cell lines. **Figure 1D** illustrated the ten cancer cell lines with the highest PABPN1 expression level. Contrastingly, in normal cells, the PABPN1 expression level in immune cells was the highest (**Figure 1C**). Additional detailed information regarding PABPN1 expression can be found in **Supplementary Table S1**. These results collectively suggested that PABPN1 was overexpressed in cancer tissues and may play a role in immune regulation processes.

### PABPN1 was a prognostic pan-cancer biomarker

**Figure 2:**
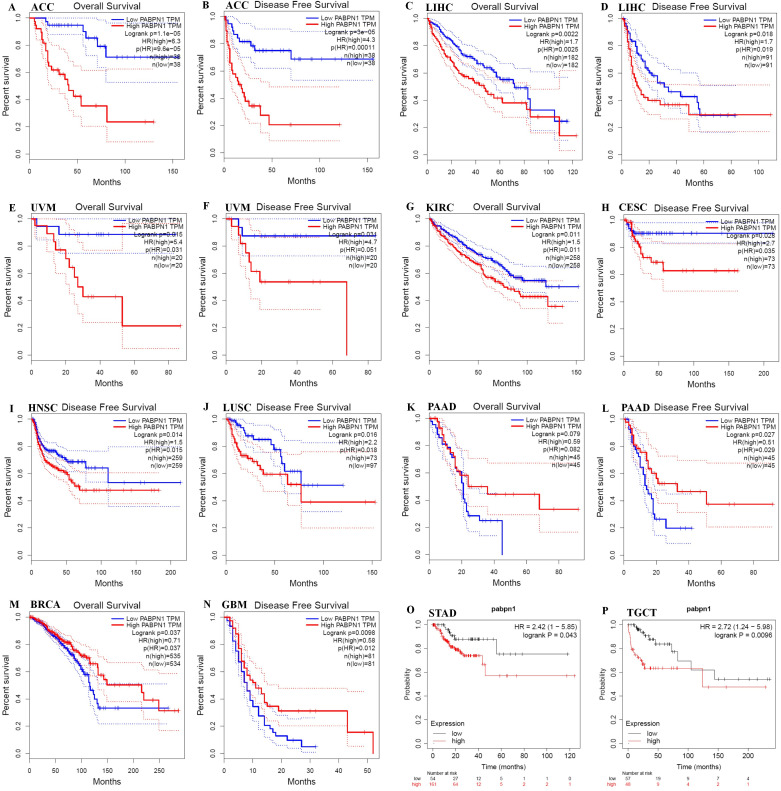
Kaplan-Meier survival curve of human cancers with high and low PABPN1 expression analyzed by the GEPIA database **(A–M)** and the Kaplan-Meier plotter database **(N–P)**. **(A–F)** High PABPN1 expression was associated with worse OS and DFS in ACC, LIHC, and UVM cohorts, respectively. **(H–J)** High PABPN1 expression was linked to worse DFS in CESC, HNSC, and LUSC cohorts. **(K, L)** High PABPN1 expression was associated with better OS and DFS in PAAD cohorts. **(M)** High PABPN1 expression was correlated with better OS in BRCA. **(N–P)** Higher PABPN1 expression was associated with poorer RFS in Myeloma, STAD and TGCT, respectively.

The prognostic significance of PABPN1 expression in human cancers was assessed through several databases. In GEPIA, higher PABPN1 expression correlated with poorer OS and DFS in ACC (n = 38, OS: HR = 6.3, P = 9.6×10^-5^; n = 38, DFS: HR = 4.3, P = 0.00011; **Figures 2A, B**) and LIHC (n = 182, OS: HR = 1.7, P = 0.0025; n = 182, DFS: HR = 1.7, P = 0.019; **Figures 2C, D**). Furthermore, elevated PABPN1 expression was associated with poorer OS and DFS in UVM (uvea lmelanoma) (n=20, OS: HR=5.4, P = 0.031, DFS: HR = 4.7, P = 0.051; **Figures 2E, F**) and KIRC (n =258, HR =1.5, P =0.011; **Figure 2G**). Additionally, patients with higher PABPN1 expression exhibited poor DFS in CESC (n = 73, HR = 2.7, P = 0.035; **Figure 2H**), LUSC (n = 97, HR = 2.2, P = 0.018; **Figure 2J**) and PAAD (n = 45, HR = 0.51, P = 0.029; **Figure 2L**). In the Kaplan-Meier plotter database, increased PABPN1 expression was linked to poorer RFS in Multiple Myeloma (n = 538, HR = 1.70, P = 0.012; **Figure 2N**), STAD (n = 215, HR = 2.42, P = 0.043; **Figure 2O**) and TGCT (n = 105, HR = 2.72, P = 0.0096; **Figure 2P**). Additional details regarding the relationship between PABPN1 and RFS analyzed by the Kaplan-Meier plotter database were provided in **Supplementary Figure S1**. Moreover, PrognoScan analysis revealed a correlation between higher PABPN1 expression and worse survival outcomes in many cancer types, including blood cancer (B-cell lymphoma), lung cancer, breast cancer, bladder cancer, esophagus cancer, brain cancer and colorectal cancer. Specific details are presented in **Supplementary Table S3**. These results collectively supported the close association of PABPN1 expression with the prognosis of many cancer types.

### The expression of PABPN1 was associated with immune and molecular subtypes in human cancers

**Figure 3:**
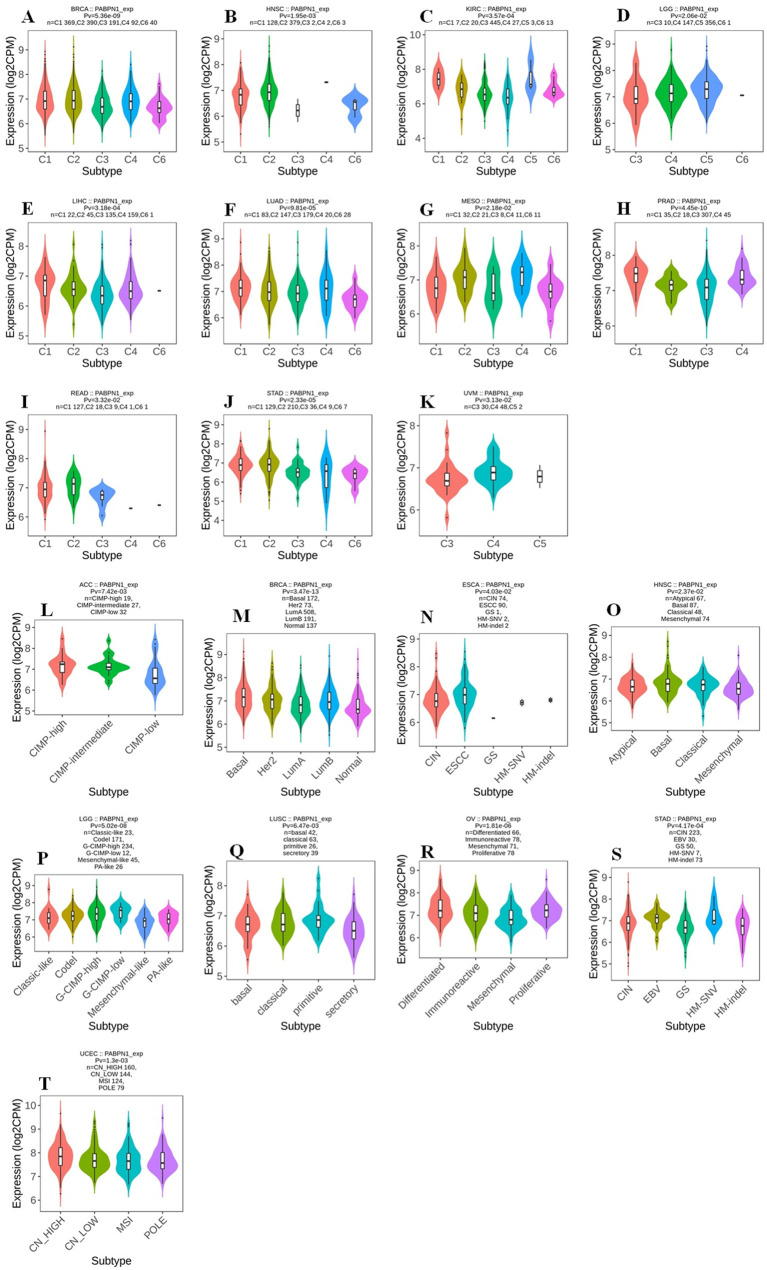
The association between PABPN1 expression and pan-cancer immune/ molecular subtypes. Immune subtypes: **(A)** BRCA, **(B)** HNSC, **(C)** KIRC, **(D)** LGG, **(E)** LIHC, **(F)** LUAD, **(G)** MESO, **(H)** PRAD, **(I)** READ, **(J)** STAD, **(K)** UVM; Molecular subtypes: **(L)** ACC, **(M)** BRCA, **(N)** ESCA, **(O)** HNSC, **(P)** LGG, **(Q)** LUSC, **(R)** OV, **(S)** STAD, **(T)** UCEC.

The TISIDB website was utilized to investigate the impact of PABPN1 expression on immune and molecular subtypes across human cancers. Immune subtypes were categorized into six types: C1 (wound healing), C2 (IFN-gamma dominant), C3 (inflammatory), C4 (lymphocyte depleted), C5 (immunologically quiet), and C6 (TGF-b dominant). Results indicated that PABPN1 expression was associated with different immune subtypes in BRCA, HNSC, KIRC, LGG (brain lower grade glioma), LIHC, LUAD, MESO, PRAD, READ, STAD, and UVM (**Figure 3**). Furthermore, PABPN1 expression varied within different immune subtypes of one cancer type. Taking KIRC for instance, PABPN1 exhibited high expression in C1 and C5 types and low expression in C3 and C4 types. Regarding different molecular subtypes of cancers, a significant correlation with PABPN1 expression was observed in ACC, BRCA, ESCA, HNSC, LGG, LUSC, OV, STAD, and UCEC (**Figure 3**). The expression of PABPN1 in different immune and molecular subtypes of other cancers was detailed in **Supplementary Figures S2** and **S3**. Based on these results, it can be concluded that PABPN1 expression varied across immune subtypes and molecular subtypes in several human cancer types.

### Relationship between PABPN1 expression and immune checkpoint genes in human cancers

**Figure 4:**
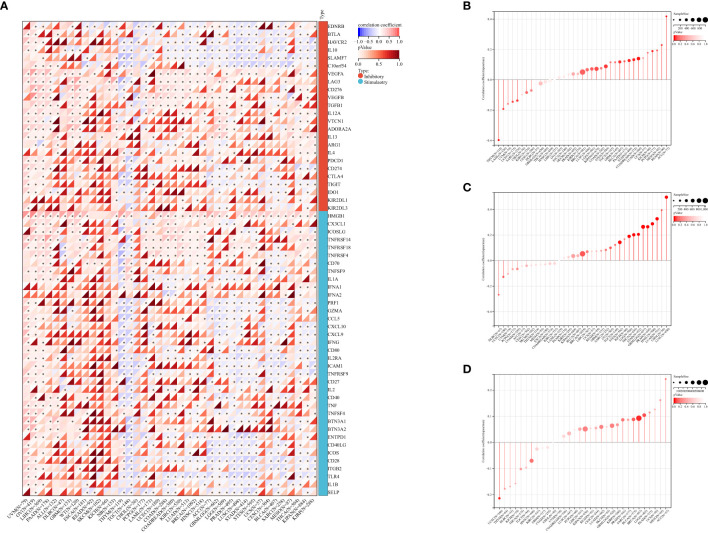
The correlation between PABPN1 expression and pan-cancer immune checkpoint genes **(A)**, TMB **(B)**, MSI **(C)**, neoantigen **(D)**, ESTIMATE score.

Studies have demonstrated the significant impact of ICP genes on immune cell infiltration and immunotherapy (33). In light of this, we delved into the connections between PABPN1 expression and ICP genes across human cancers, aiming to uncover the potential role of PABPN1 in immunotherapy. Among the 60 ICP genes studied, associations with PABPN1 expression were identified in many cancer types, including OV, UVM, LIHC, PAAD, ALL, DLBC, GBM, WT, THYM, TGCT, CHOL, PCPG, LAML, UCEC, COAD, KIRC, LUAD, BRCA, HNSC, ACC, GBMLGG, LGG, PRAD, LUSC, STAD, STES, UCS, CESC, BLCA, SARC, MESO, THCA, KIPAN, and KIRP (**Figure 4A**). Particularly in OV, UVM, LIHC, PAAD, ALL, and WT, PABPN1 expression showed a positive correlation with immune checkpoint genes. Notably, in OV, 47 out of the 60 immune checkpoint genes exhibited connections with PABPN1 expression. This suggested that PABPN1 might play a role in coordinating the activity of these ICP genes within different signal transduction pathways, positioning it as a potential immunotherapy target. Furthermore, high PABPN1 expression in certain cancers, especially OV, may serve as an indicator of favorable therapeutic efficacy in immunotherapies targeting ICP genes. The intricate relationship between PABPN1 expression and immune checkpoint genes in many cancer types warranted further investigation. In summary, our hypothesis suggested that PABPN1 could serve as a potential pan-cancer biomarker or a novel immunotherapy target, with the potential to predict the response to immunotherapy.

### The correlation between PABPN1 expression and TMB, MSI, neoantigen, and ESTIMATE

To investigate PABPN1’s role in the immune mechanisms and responses within the TME, we conducted an analysis of the correlations between PABPN1 expression and TMB, MSI, and neoantigens. TMB, MSI, and neoantigens in the tumor microenvironment are recognized as indicators of antitumor immunity and can potentially predict the efficacy of tumor immunotherapy (34–36).

Our analysis revealed positive correlations between PABPN1 expression and TMB, MSI, and neoantigens in several cancer types, including ACC, BRCA, LUSC, KIPAN, LGG, STES, BLCA, PAAD, LUAD, MESO, and READ (**Figures 4B–D**). Subsequently, we delved into the ESTIMATE score of PABPN1 across many cancer types (**Supplementary Figure S4**). **Supplementary Table S4** provided detailed associations between PABPN1 expression and the three ESTIMATE categories. Notably, strong negative correlations were observed in ACC, BLCA, BRCA, CESC, COAD, GBM, HNSC, KIRP, LGG, LUAD, LUSC, OV, PRAD, STES, STAD, THCA, and TGCT. The obtained results provided additional confirmation supporting the hypothesis that PABPN1 may impact antitumor immunity through the regulation of the composition and immune mechanisms within the tumor microenvironment.

### PABPN1 correlated with immune cell infiltration in the TME in human cancers

**Figure 5:**
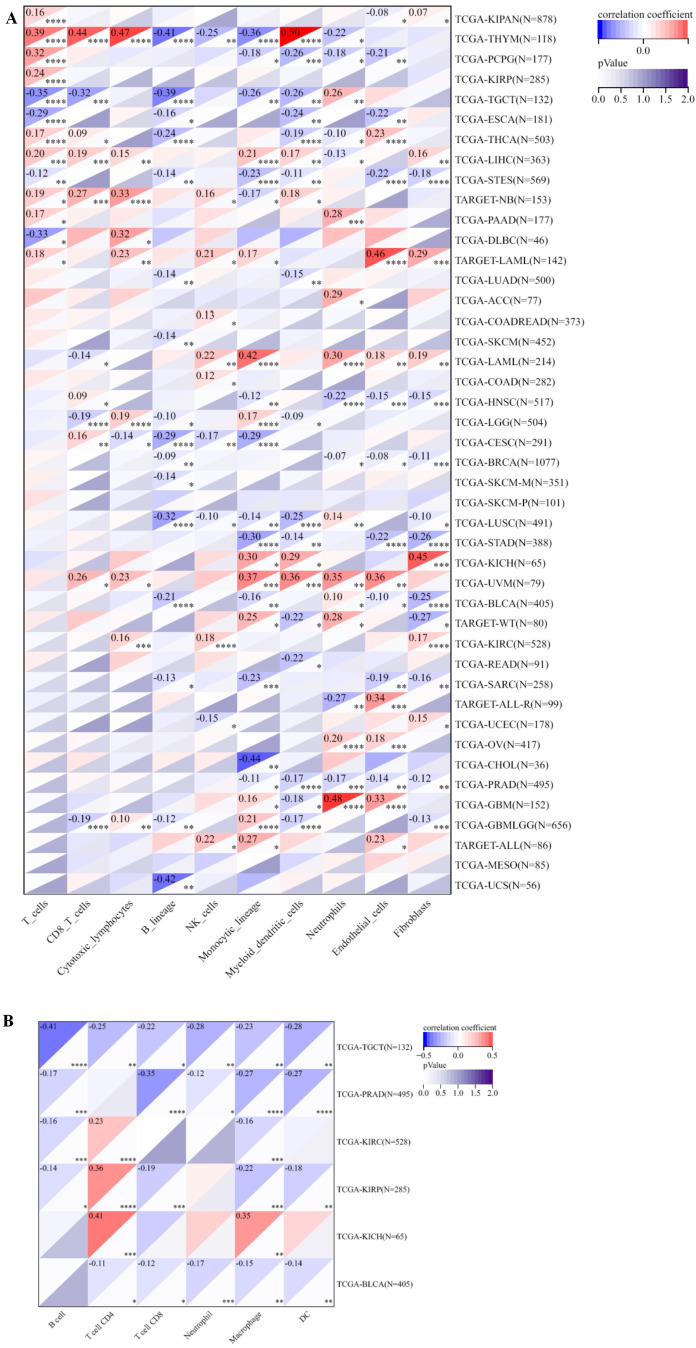
The relationship between PABPN1 expression and infiltrating immune cells of human cancers and urogenital cancers. **(A)** The relationship between PABPN1 expression level and infiltrating levels of T cells, CB8+ T cells, cytotoxic lymphocytes, B cell lineages, natural killer cells, monocytic cells, myeloid dendritic cells, neutrophils, endothelial cells and fibroblasts in 45 cancer types. **(B)** The relationship between PABPN1 expression level and infiltrating levels of B cells, CD4+ T cells, CB8+ T cells, neutrophils, macrophages and dendritic cells in six urogenital cancers. *P < 0.05; **P < 0.01; ***P < 0.001; ****P < 0.0001.

PABPN1’s correlation with immune cell infiltration in the TME across different human cancers was investigated. After establishing differential PABPN1 expression in distinct immune subtypes, the exploration of potential correlations with immune cell infiltration revealed significant associations in 42 cancer types. PABPN1 expression displayed strong correlations with immune cell types, including CD8+ T cells in 11 cancer types, cytotoxic lymphocytes in 10 cancer types, B cells in 16 cancer types, natural killer cells in 11 cancer types, monocytic cells in 23 cancer types, myeloid dendritic cells in 19 cancer types, neutrophils in 18 cancer types, endothelial cells in 18 cancer types, and fibroblasts in 17 cancer types (**Figure 5A**). These findings were consistent with the results obtained from the TIMER database, detailed in **Supplementary Figure S5**.

Further analysis focused on the connection between PABPN1 expression and immune cell infiltration in six urogenital cancers. The findings indicated that PABPN1 expression was associated with the infiltration levels of immune cell types in specific urogenital cancers (**Figure 5B**). In TGCT, PABPN1 expression correlated with the infiltration levels of B cells, CD4+ T cells, CD8+ T cells, neutrophils, macrophages, and dendritic cells. In KIRC, the correlation was observed with B cells, CD8+ T cells, neutrophils, macrophages, and dendritic cells. In KIRP, PABPN1 expression was correlated with B cells, CD4+ T cells, CD8+ T cells, macrophages, and dendritic cells. In KICH, the correlation was observed with CD4+ T cells and macrophages. Lastly, in BLCA, PABPN1 expression was associated with CD4+ T cells, CD8+ T cells, neutrophils, macrophages, and dendritic cells. To validate these findings, the relationship was verified using EPIC, and most results from this database were consistent with the initial observations (**Supplementary Figure S6**). In summary, these results strongly supported the notion that PABPN1 expression influenced immune cell infiltration in urogenital cancers.

### PABPN1 correlated with immune cell infiltration and their gene markers in TME in LIHC, BLCA and TGCT

In the analysis of LIHC, BLCA, and TGCT using the TIMER database, we delved deeper into the correlation between PABPN1 expression and marker subsets of immune cells, adjusting for purity to mitigate biases introduced by clinical samples. In LIHC (n = 363), BLCA (n = 405), and TGCT (n = 132), we aimed to elucidate the potential immune functions of PABPN1 across different cancer types. As outlined in **Table 1**, PABPN1 demonstrated a close association with most included marker genes of B cells, CD8+ T cells, monocytes, tumor-associated macrophages (TAMs), M2 macrophages, and Th2 cells in LIHC and TGCT. However, in BLCA, after purity adjustment, PABPN1 expression exhibited a less robust connection, being correlated mainly with marker genes of CD8+ T cells, monocytes, M2 macrophages, and Th17. The varying relationships between PABPN1 expression and gene markers of immune cell infiltration in LIHC, BLCA, and TGCT may contribute to the distinct survival outcomes observed in different cancer types. The findings suggested that PABPN1 may exert regulatory influences on immune cell functions, potentially engaging in mechanisms similar to those involving marker genes.

**Table 1:** Correlation between PABPN1 and relate genes and markers of immune cells analyzed by TIMER.

Description	Gene markers	LIHC				BLCA				TGCT			
		None		Purity		None		Purity		None		Purity	
		Cor	P	Cor	P	Cor	P	Cor	P	Cor	P	Cor	P
CD8+ T cell	CD8A	0.125	*	0.218	***	-0.105	*	0.080	0.125	-0.378	***	-0.360	***
	CD8B	0.130	*	0.223	***	-0.014	0.773	0.131	*	-0.369	***	-0.335	***
T cell (general)	CD3D	0.211	***	0.318	***	-0.127	**	0.081	0.120	-0.343	***	-0.332	***
	CD3E	0.158	**	0.290	***	-0.168	**	0.051	0.326	-0.373	***	-0.373	***
	CD2	0.160	**	0.283	***	-0.153	**	0.070	0.182	-0.337	***	-0.319	***
B cell	CD19	0.196	***	0.247	***	-0.202	***	-0.077	0.139	-0.421	***	-0.422	***
	CD79A	0.165	**	0.264	***	-0.233	***	-0.091	0.081	-0.390	***	-0.386	***
Monocyte	CD86	0.228	***	0.354	***	-0.240	***	-0.051	0.327	-0.278	**	-0.232	**
	CSF1R	0.126	*	0.237	***	-0.290	***	-0.106	*	-0.318	***	-0.278	**
TAM	CCL2	0.102	*	0.191	***	-0.221	***	-0.063	0.226	0.100	0.222	0.137	0.099
	CD68	0.148	**	0.225	***	-0.204	***	-0.068	0.196	-0.387	***	-0.358	***
	IL10	0.202	***	0.301	***	-0.237	***	-0.067	0.201	-0.362	***	-0.337	***
M1 Macrophage	NOS2	-0.032	0.539	-0.035	0.512	0.021	0.667	0.102	*	0.008	0.918	-0.053	0.527
	IRF5	0.337	***	0.328	***	0.024	0.634	-0.001	0.990	-0.279	**	-0.233	**
	PTGS2	0.113	*	0.209	***	-0.098	*	-0.047	0.370	-0.009	0.908	-0.051	0.540
M2 Macrophage	CD163	-0.017	0.750	0.053	0.327	-0.296	***	-0.121	*	-0.011	0.894	0.021	0.799
	VSIG4	-0.014	0.794	0.064	0.237	-0.283	***	-0.108	*	0.003	0.970	0.022	0.787
	MS4A4A	0.007	0.891	0.099	0.067	-0.259	***	-0.060	0.248	-0.163	*	-0.116	0.161
Neutrophils	CEACAM8	0.009	0.870	0.036	0.506	0.023	0.637	-0.021	0.682	0.208	*	0.218	**
	ITGAM	0.222	***	0.286	***	-0.259	***	-0.069	0.186	-0.167	*	-0.116	0.164
	CCR7	0.096	0.065	0.196	***	-0.008	0.879	0.068	0.191	-0.160	*	-0.076	0.358
Natural killer cell	KIR2DL1	0.006	0.902	-0.006	0.910	-0.122	*	-0.021	0.687	-0.151	0.066	-0.076	0.357
	KIR2DL3	0.165	**	0.198	***	-0.076	0.126	0.035	0.507	-0.197	*	-0.133	0.107
	KIR2DL4	0.169	**	0.209	***	-0.052	0.294	0.089	0.089	-0.118	0.150	-0.020	0.806
	KIR3DL1	0.068	0.189	0.081	0.134	-0.033	0.510	0.065	0.212	-0.132	0.108	-0.045	0.589
	KIR3DL2	0.045	0.382	0.097	0.072	-0.024	0.624	0.084	0.109	-0.250	**	-0.191	*
	KIR3DL3	0.065	0.213	0.070	0.198	0.055	0.266	0.096	0.067	-0.282	***	-0.239	**
	KIR2DS4	0.059	0.260	0.067	0.211	-0.066	0.185	0.031	0.551	-0.182	*	-0.110	0.185
Dendritic cell	HLA-DPB1	0.107	*	0.191	***	-0.262	***	-0.079	0.131	-0.389	***	-0.370	***
	HLA-DQB1	0.096	0.064	0.187	***	-0.219	***	-0.026	0.620	-0.268	**	-0.233	**
	HLA-DRA	0.095	0.066	0.174	**	-0.232	***	-0.053	0.307	-0.402	***	-0.391	***
	HLA-DPA1	0.094	0.072	0.180	**	-0.228	***	-0.057	0.276	-0.422	***	-0.408	***
	CD1C	0.096	0.065	0.159	**	-0.243	***	-0.129	*	-0.324	***	-0.306	***
	NRP1	0.337	***	0.357	***	-0.114	*	-0.017	0.749	-0.101	0.218	-0.134	0.106
	ITGAX	0.323	***	0.440	***	-0.212	***	-0.007	0.893	-0.290	***	-0.249	**
Th1	TBX21	0.092	0.075	0.179	**	-0.108	*	0.082	0.116	-0.380	***	-0.377	***
	STAT4	0.200	***	0.244	***	-0.172	***	0.005	0.919	-0.253	**	-0.213	**
	STAT1	0.233	***	0.255	***	-0.088	0.076	0.059	0.262	-0.471	***	-0.450	***
	IFNG	0.177	**	0.244	***	-0.061	0.216	0.098	0.060	-0.308	***	-0.265	**
	TNF	0.259	***	0.355	***	0.002	0.967	0.105	0.044	-0.237	**	-0.188	*
	IL12A	0.435	***	0.461	***	0.018	0.724	0.098	0.062	-0.194	*	-0.196	*
	IL12B	0.109	*	0.154	**	-0.129	**	0.001	0.987	-0.490	***	-0.479	***
Th2	GATA3	0.199	***	0.322	***	0.190	***	0.088	0.091	0.214	**	0.185	*
	STAT6	0.163	**	0.130	*	0.072	0.149	0.022	0.680	0.167	*	0.138	0.096
	STAT5A	0.290	***	0.326	***	-0.217	***	-0.124	0.018	-0.350	***	-0.312	***
	IL13	0.135	**	0.120	*	-0.058	0.241	0.020	0.701	-0.005	0.952	-0.020	0.812
Tfh	BCL6	0.347	***	0.356	***	0.088	0.074	0.058	0.271	-0.034	0.682	-0.035	0.671
	IL21	0.027	0.601	0.066	0.223	-0.031	0.533	0.042	0.425	-0.359	***	-0.325	***
Th17	STAT3	0.064	0.218	0.088	0.104	-0.143	**	-0.055	0.297	-0.081	0.325	-0.087	0.294
	IL17A	0.090	0.083	0.101	0.062	0.077	0.123	0.105	*	-0.134	0.103	-0.106	0.199
Treg	FOXP3	0.083	0.111	0.122	*	-0.105	*	0.086	0.098	-0.203	*	-0.133	0.109
	CCR8	0.233	***	0.291	***	-0.122	*	0.030	0.564	0.060	0.469	0.109	0.189
	STAT5B	0.239	***	0.202	***	0.035	0.481	0.033	0.527	-0.506	***	-0.493	***
	TGFB1	0.276	***	0.353	***	-0.149	**	-0.065	0.213	-0.283	***	-0.283	**
T cell exhaustion	PDCD1	0.290	***	0.383	***	-0.132	**	0.070	0.181	-0.348	***	-0.333	***
	CTLA4	0.299	***	0.409	***	-0.128	**	0.077	0.141	-0.309	***	-0.270	**
	LAG3	0.267	***	0.317	***	-0.076	0.125	0.122	*	-0.246	**	-0.187	*
	HAVCR2	0.244	***	0.378	***	-0.243	***	-0.044	0.395	-0.215	**	-0.148	0.074
	GZMB	0.134	*	0.203	***	-0.145	**	0.055	0.294	-0.043	0.601	0.073	0.382

*P < 0.05; **P < 0.01; ***P < 0.001.

### PABPN1 expression in different clinical subgroups of urogenital cancers

**Figure 6:**
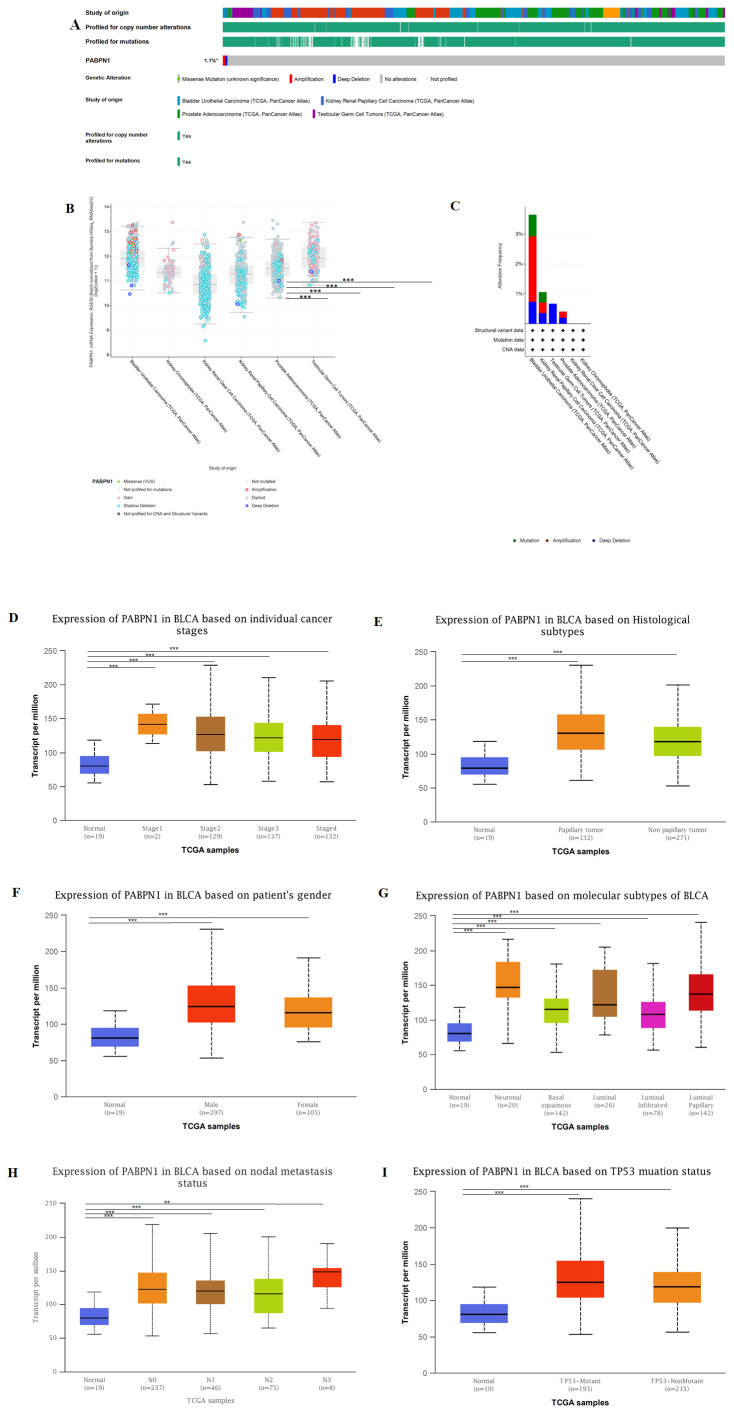
PABPN1 genomic alterations and differential expression in urogenital cancers analyzed by the cBioPortal and UALCAN database. **(A)** OncoPrint of PABPN1 gene alterations in the cancer cohort. Different colors indicate different genetic alteration types, with amplification being predominant. **(B)** Main types of PABPN1 gene alterations observed in cancer groups. **(C)** Details of PABPN1 gene alteration types within the cancer cohort. **(D–I)** PABPN1 expression in bladder cancer across specific clinical subgroups, encompassing cancer stages **(D)**, histological subtypes **(E)**, patient sex **(F)**, molecular subtypes **(G)**, nodal metastasis status **(H)**, and TP53 mutation status **(I)**. **P < 0.01; ***P < 0.001.

The cBioPortal website was utilized to analyze genomic alterations of PABPN1 in urogenital cancers. Results indicated that genomic alterations of PABPN1 occurred in 1.1% of patients (**Figure 6A** and **Supplementary Figure S7**). The types of PABPN1 gene alterations were diverse, leading to changes in gene expression (**Figure 6B**). Copy number variation (CNV) predominantly occurred in BLCA, with no CNV observed in KICH and KIRC (**Figure 6C**). Subsequently, the UALCAN database was employed to investigate PABPN1 expression in urogenital cancers with different clinical characteristics. Using BLCA as an example, significant differential expression of PABPN1 was observed across various cancer stages, histological subtypes, patient sex, molecular subtypes, nodal metastasis status, and TP53 mutation status of BLCA (**Figures 6D–I**). PABPN1 expression in five other urogenital cancers with different cancer stages was presented in **Supplementary Figure S7**. All these findings collectively suggested that PABPN1 may play a crucial role in the onset and progression of cancer in urogenital tissues.

### Analysis of PABPN1 coexpression networks

**Figure 7:**
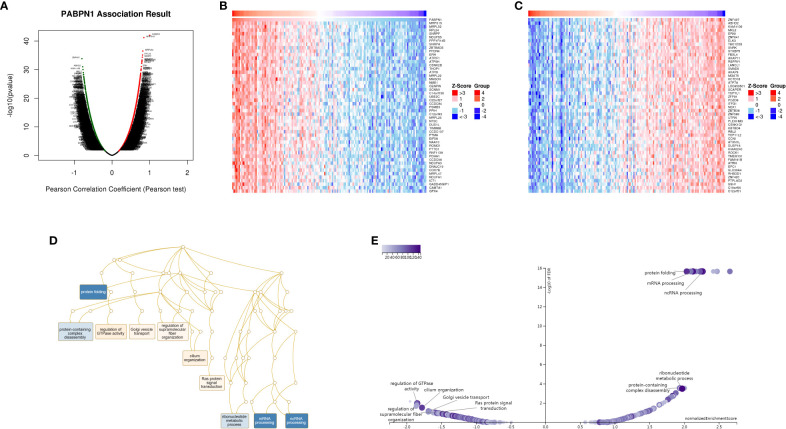
PABPN1 coexpression genes in TGCT analyzed by the LinkedOmics database. **(A)** Highly correlated genes of PABPN1 tested by Pearson test in TGCT cohort. **(B, C)** Top 50 positive coexpression genes **(B)** and negative coexpression genes **(C)** of PABPN1 in heat map in TGCT; **(D)** Directed acyclic graph of PABPN1 GO analysis (biological process) in TGCT cohort. **(E)** Volcano plot of PABPN1 KEGG pathways in TGCT.

The preceding findings highlighted a substantial association between PABPN1 and cancer prognosis and immunity. To further elucidate the potential functional role of PABPN1 in tumor tissue, we investigated PABPN1 coexpression networks using the LinkedOmics database. TGCT was chosen as an illustrative example to showcase the potential effects (**Figure 7A**). **Supplementary Table S5** presented all coexpressed genes positively or negatively linked to PABPN1. The top 50 genes, both positively and negatively correlated with PABPN1, were displayed in a heatmap (**Figures 7B, C**). Among these, MRPS15, MRPL52, and RPL24 exhibited the strongest associations with PABPN1 expression (r = 0.844, 0.817, 0.808, and p = 6.735e-42, 3.149e-37, 7.687e-36, respectively).

Subsequently, GSEA was employed to identify the primary GO terms of PABPN1 coexpression genes. The analysis revealed that PABPN1 and its coexpression genes were predominantly involved in the regulation of RNA processes, protein complex disassembly, GTPase activity, and Golgi vesicle transport (**Figure 7D**) within the biological process categories of GO. The molecular function (MF) and cell component (CC) analyses of PABPN1 coexpression genes were presented in **Supplementary Figure S9**. Furthermore, KEGG pathway analysis showed that the coexpressed genes were enriched in oxidative phosphorylation, spliceosome, and glutathione metabolism (**Figure 7E**).

### Experimental validation of bioinformatics results

**Figure 8:**
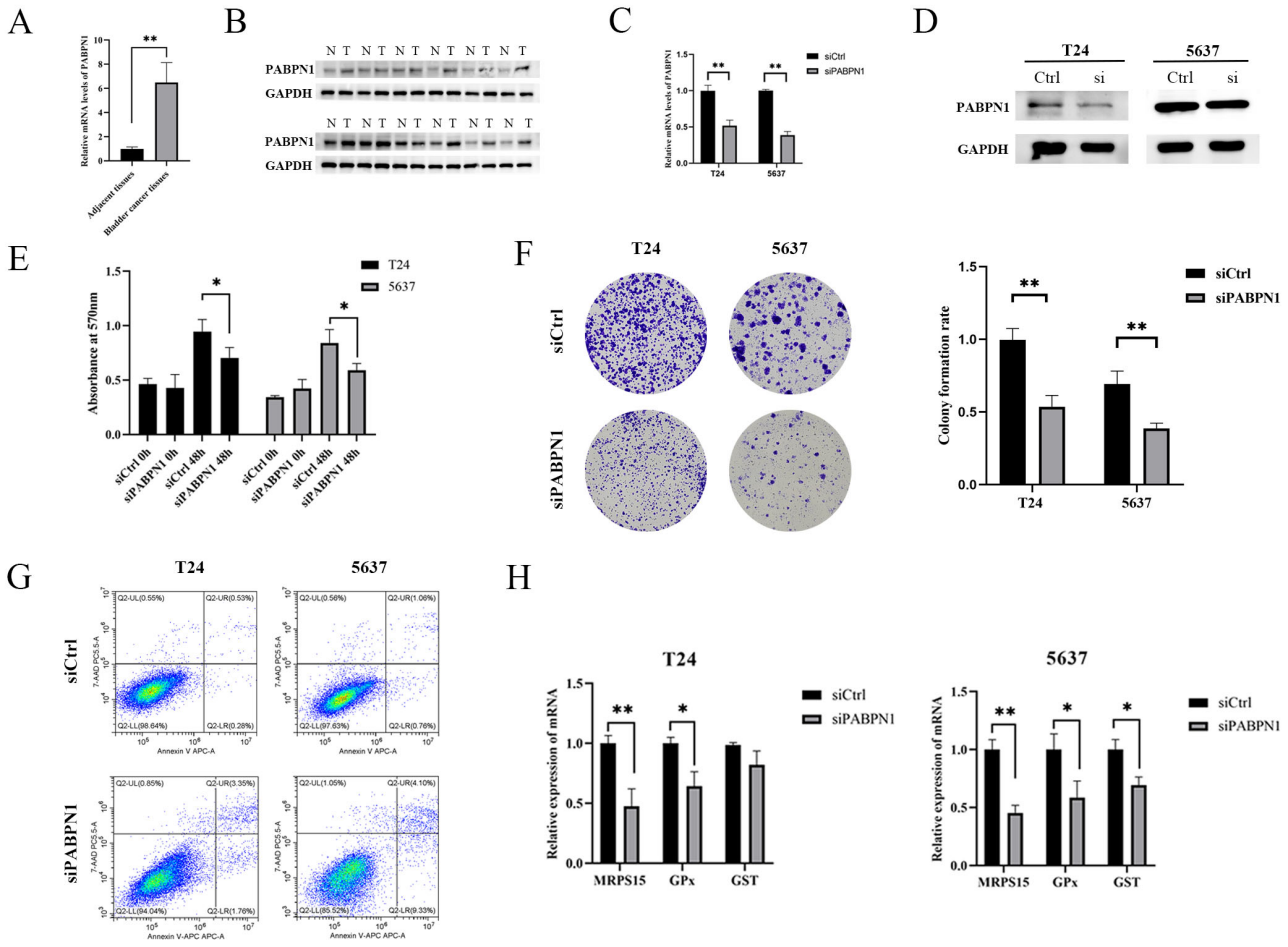
PABPN1 knockdown inhibits cell proliferation. **(A)** mRNA expression of PABPN1 in bladder cancer and para-carcinoma tissues (All were 5 samples). **(B)** Protein expression of PABPN1 in bladder cancer and para-carcinoma tissues. **(C)** mRNA level of PABPN1 in T24 and 5637 cells treated with siPABPN1. **(D)** protein level of PABPN1 in T24 and 5637 cells treated with siPABPN1. **(E)** Viability of cells tested by MTT. **(F)** Colony formation assay conducted in PABPN1-knockdown HCCLM3 cells. **(G)** Detection of apoptosis by flow cytometry treated with siPABPN1. **(H)** Effect of PABPN1 knockdown on gene expression (MRPS15, GPx and GST). *P < 0.05; **P < 0.01.

The expression of PABPN1 in BLCA, LIHC and BRCA tissues was significantly higher than that in para-carcinoma tissues (**Figure 8 A, B**; **Supplementary Figures S11A–D**). To investigate the functional role of PABPN1, we transfected T24, 5637, HLF and MCF-7 cells with PABPN1 siRNAs. RT-qPCR confirmed effective knockdown (**Figure 8C**; **Supplementary Figures S11E, G**), and Western blot analyses validated this finding (**Figure 8D**; **Supplementary Figures S11F, H**). For cell proliferation studies, MTT and colony formation assays revealed that reduced PABPN1 expression decreased T24, 5637, HLF and MCF-7 cells viability (**Figure 8E**; **Supplementary Figures S11I, K**) and significantly reduced colony numbers (**Figures 8F**; **Supplementary Figures S11J, L**). Additionally, flow cytometric analysis revealed that PABPN1 downregulation promoted apoptosis in these cells (**Figures 8G**; **Supplementary Figures S11M, O**). The mRNA expression levels of MRPS15 and GPx (Glutathione peroxidase) decreased notably in T24, 5637, HLF and MCF-7 cells after siPABPN1 transfection (**Figure 8H**; **Supplementary Figures S11N, P**). Collectively, the results substantiated our assumption that PABPN1 was a potential pan-cancer biomarker.

## Discussion

PABPN1, formerly recognized as a versatile facilitator of nuclear polyadenylation, engages in several key functions, such as associating with the elongating poly(A) tail and interacting with poly(A) polymerase, regulating poly(A) tail length, and influencing alternative cleavage and polyadenylation processes (37, 38). Despite being categorized as a nuclear poly(A)-binding protein, PABPN1 was observed in the peri-nuclear region of the cytoplasm, suggesting a potential role as a mediator shuttling between the nucleus and cytoplasm (39). Several decades ago, mutations in the PABPN1 gene were identified as the underlying cause of Oculopharyngeal Muscular Dystrophy (OPMD), impacting a specific group of skeletal muscles (40). Earlier research has established the critical role of PABPN1 in major zygotic genome activation and its regulatory function in the expression of cell fate-determining factors during the preimplantation stage in mouse embryos (41). In this study, we revealed PABPN1’s pivotal involvement in oncogenesis and the progression of tumors across many types of cancer.

In the initial phase of our investigation, we employed the TIMER, GEPIA, and BioGPS databases to assess the expression levels of PABPN1 in both cancer and normal tissues. The findings revealed a notably elevated expression of PABPN1 across many cancer types. These outcomes suggested that PABPN1 may played a significant role in fostering oncogenesis and advancing tumor progression in human cancers. Besides, DNA aberrant methylation was recognized as a common contributor to the dysregulation of tumor-associated gene expression. In our study, we delved into the Methylation-Expression correlation for pabpn1 across different cancers using the DNMIVD database (42, 43) (refer to **Supplementary Figure S10**). The outcomes revealed robust positive correlations in several cancer types, including BLCA, BRCA, CESC, COAD, LIHC, KIRC, KIRP, and SAR. Investigating abnormal PABPN1 methylation in the context of pan-cancer research holds significant promise as a novel direction for future research endeavors.

Subsequently, we delved into the correlation between PABPN1 expression and prognosis. In numerous cancer types, including ACC, CESC, GBM, KIRC, LIHC, LUSC, PAAD, and UVM, heightened PABPN1 expression corresponded to a poorer prognosis. This substantiated PABPN1’s potential as a pan-cancer prognostic biomarker. Further investigation into PABPN1 expression across different immune subtypes and molecular subtypes of human cancers, using the TISIDB website, unveiled significant variations in PABPN1 expression in many cancer types such as BRCA, HNSC, LGG, STAD, suggesting its potential role as a diagnostic pan-cancer biomarker and involvement in immune regulation. Additionally, our findings demonstrated noteworthy differences in PABPN1 expression across clinical subgroups of urogenital cancers, aligning with prior research highlighting varying PABPN1 expression in different stages of prostate cancer (11). Subsequent exploration focused on the association between PABPN1 expression and immune cell infiltration in six urogenital cancers, revealing PABPN1’s presence across diverse clinical characteristics. This implied a potential role for PABPN1 in the growth and progression of these cancers.

TILs within the tumor microenvironment have been established as an autonomous predictor of both cancer patient prognosis and the effectiveness of immunotherapeutic interventions (44). Our investigation revealed that PABPN1 expression did not consistently exhibit a negative correlation with TILs across different human cancers. These results suggested that PABPN1 may play distinct roles in immune regulation in many cancer types. The correlations with TMB, MSI, neoantigens, and ESTIMATE scores underscore the potential significance of PABPN1 in shaping the immune landscape of various cancers, contributing to our understanding of its role in the broader context of cancer immunotherapy. These correlations supported the hypothesis that PABPN1 may exert an influence on antitumor immunity by regulating the composition and immune mechanisms within the tumor microenvironment. Nevertheless, additional experimental research was required to substantiate its functional role. Additionally, PABPN1 exhibited a strong correlation with a majority of the marker genes associated with B cells, CD8+ T cells, monocytes, TAMs, M2 macrophages, and Th2 cells. This emphasized the significance of PABPN1 in shaping the immune microenvironment and hinted at its potential involvement in modulating immune responses within the context of cancer in both LIHC and TGCT.

*In vitro*, we performed functional experiments to examine the role of PABPN1 in BLCA, LIHC and BRCA progression. The downregulation of PABPN1 expression was found to suppress the proliferation of T24, 5637, HLF and MCF-7 cells while promoting apoptosis. These findings underscored the strong association between elevated PABPN1 expression and adverse outcomes in cancer patients, highlighting the potential significance of PABPN1 in driving cancer progression. The induction of apoptosis following PABPN1 knockdown may be attributed to several mechanisms. First, PABPN1 regulates mRNA stability and translation, and its depletion may destabilize mRNAs encoding anti-apoptotic proteins, such as BCL-2l2 (12),CDK2, CDK6 and CDKN1A (45), leading to increased apoptosis. Second, PABPN1 influences alternative polyadenylation (APA), and its knockdown may result in the production of truncated or non-functional protein isoforms that promote apoptosis (46). Third, the observed reduction in GPx expression following PABPN1 knockdown suggests increased oxidative stress, which can trigger intrinsic apoptosis pathways (47). Finally, mitochondrial dysfunction induced by PABPN1 depletion may lead to the release of cytochrome c and activation of the caspase cascade, further promoting apoptosis.

Despite conducting a comprehensive and systematic analysis of PABPN1 and utilizing different databases for cross-verification, this study has certain limitations. Firstly, disparities in microarray and sequencing data across different databases lacked consistency in granularity and specificity, potentially introducing systematic bias. Secondly, the validation of our findings on the potential functions of PABPN1 required *in vivo*/*in vitro* experiments to enhance the credibility of our results. Thirdly, despite our conclusion that PABPN1 expression was significantly correlated with immune cell infiltration and cancer prognosis, there was a lack of direct evidence demonstrating PABPN1’s direct influence on prognosis through participation in immune infiltration. Further study was required to elucidate the exact pathway of PABPN1’s participation in immune regulation and its specific influence on cancer prognosis. Hence, it is essential to undertake a prospective study focusing on PABPN1 expression and its involvement in immune infiltration in human cancers. Additionally, the successful development and thorough testing of a novel antitumor immunotherapy drug targeting PABPN1 should be pursued. This approach holds the potential to enhance our understanding of PABPN1’s role in cancer immunity and could lead to the creation of effective therapeutic strategies.

## Data Availability

The original contributions presented in the study are included in the article/**Supplementary Material**. Further inquiries can be directed to the corresponding author.
