# Supplementary figures and images for: PABPN1 as a pan-cancer biomarker: prognostic significance and association with tumor immune microenvironment
Source: Front Immunol. 2025 Jun 18;16:1553527. doi: 10.3389/fimmu.2025.1553527 (PMC12213777; doi:10.3389/fimmu.2025.1553527)

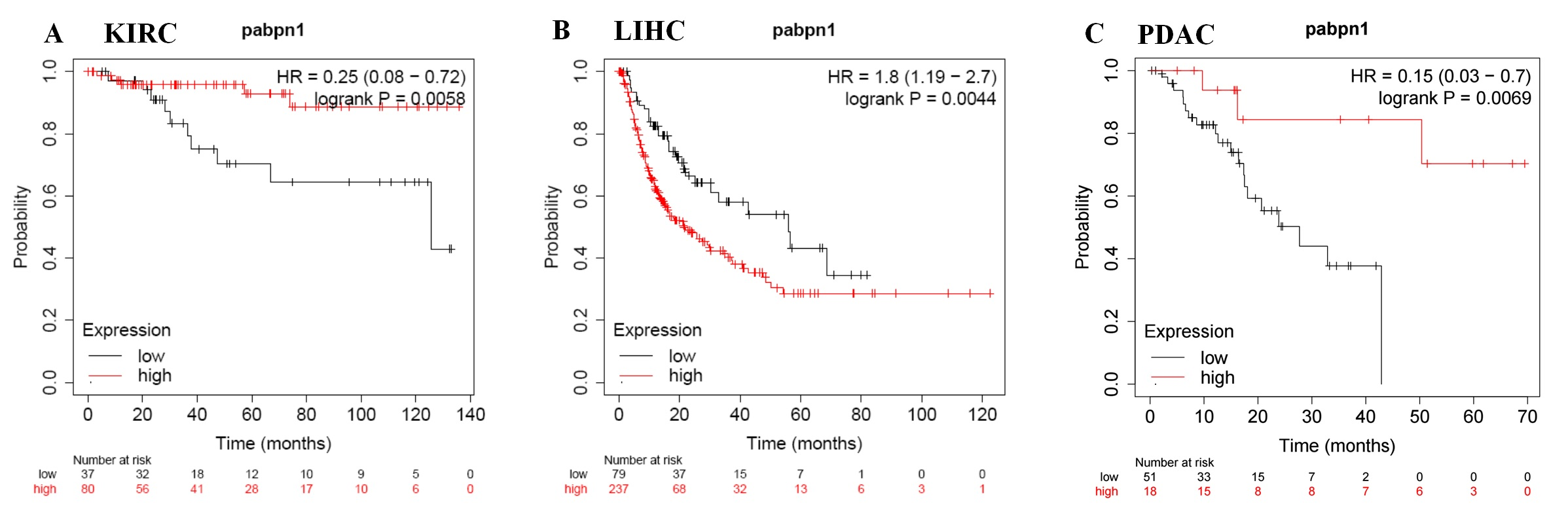

Supplement: Supplementary file 1 [file Image1.tif]

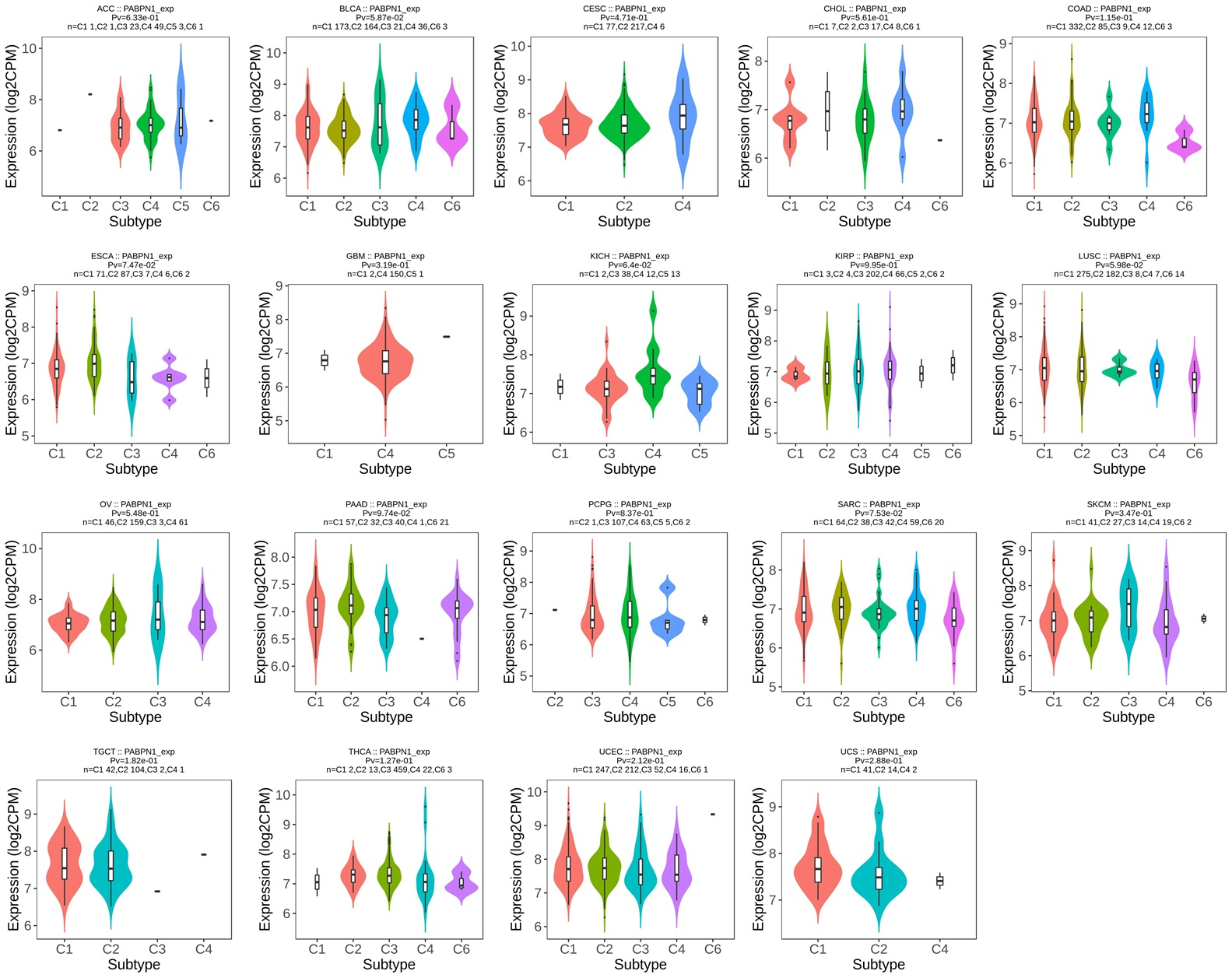

Supplement: Supplementary file 2 [file Image2.tif]

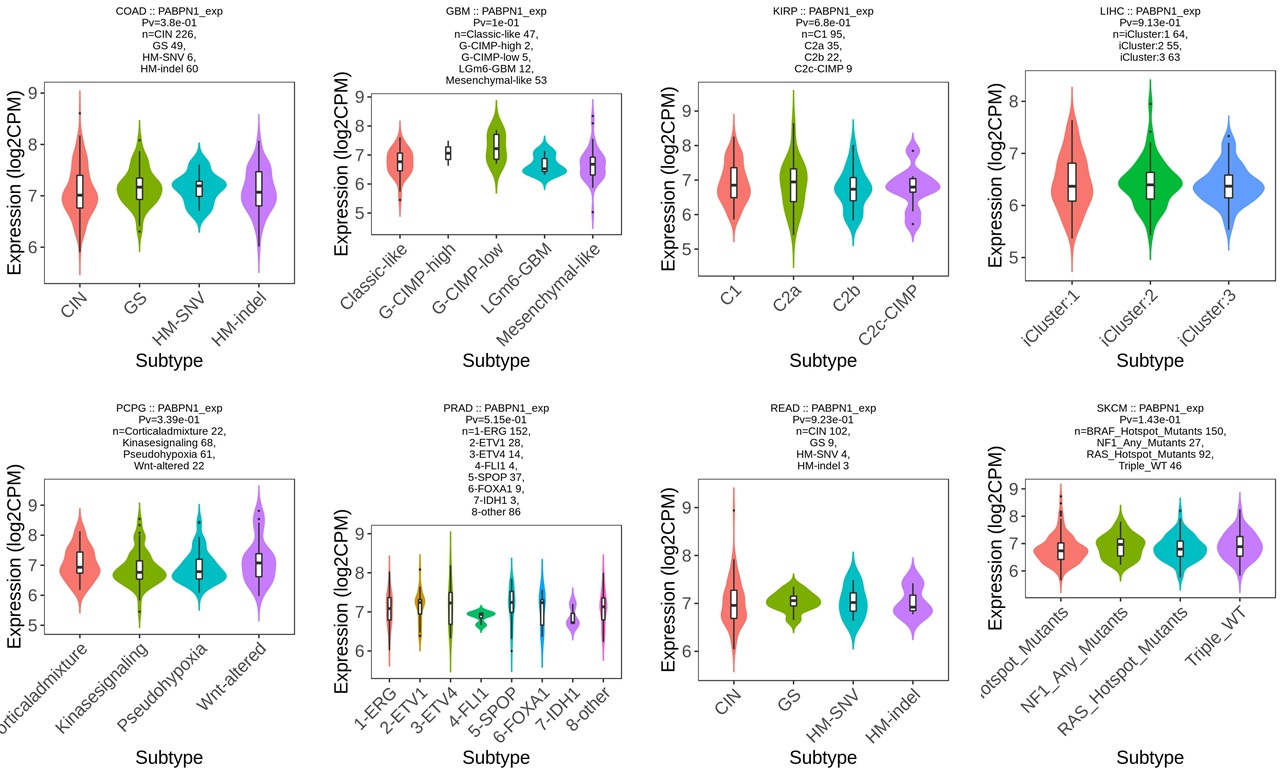

Supplement: Supplementary file 3 [file Image3.tif]

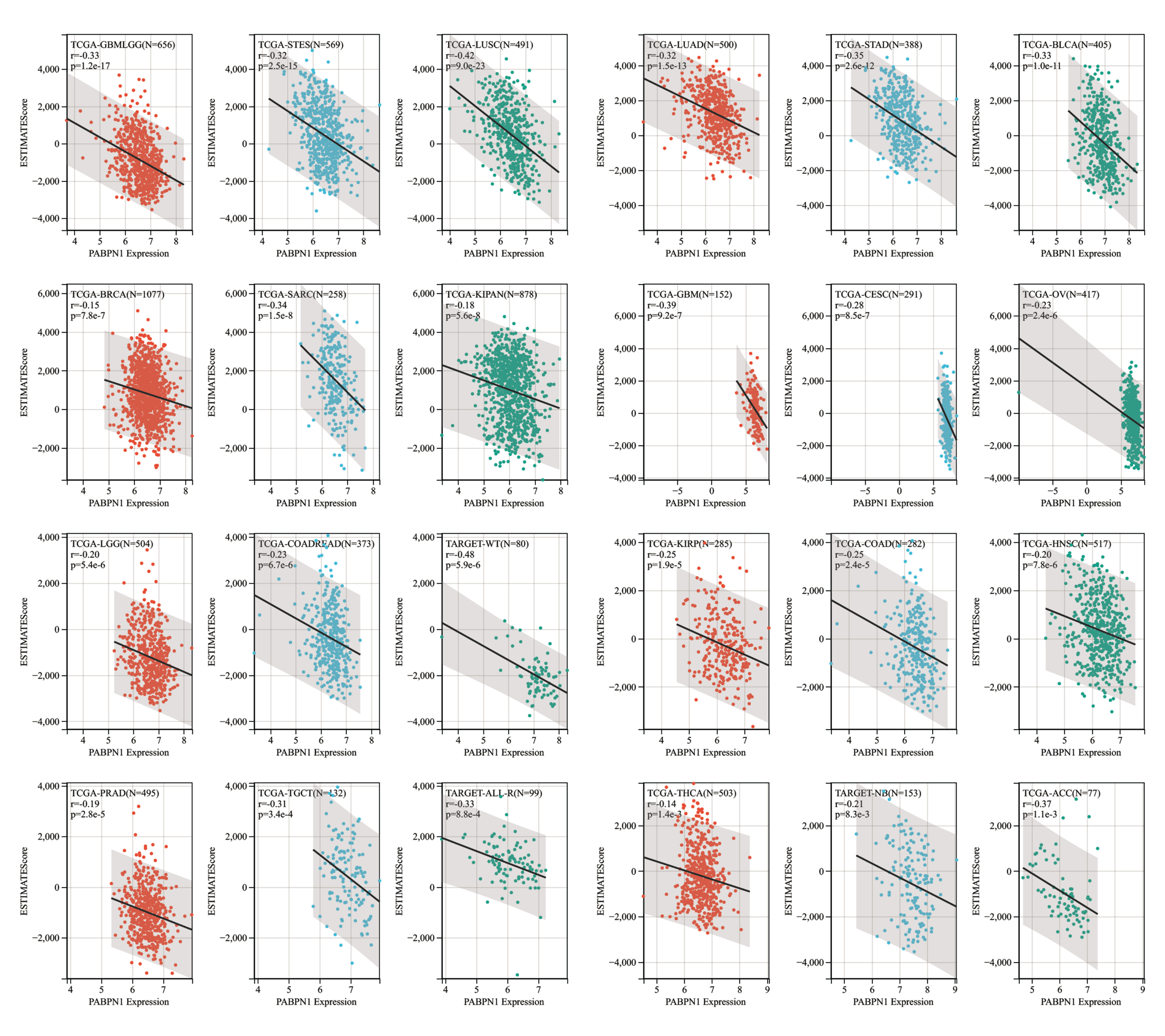

Supplement: Supplementary file 4 [file Image4.tif]

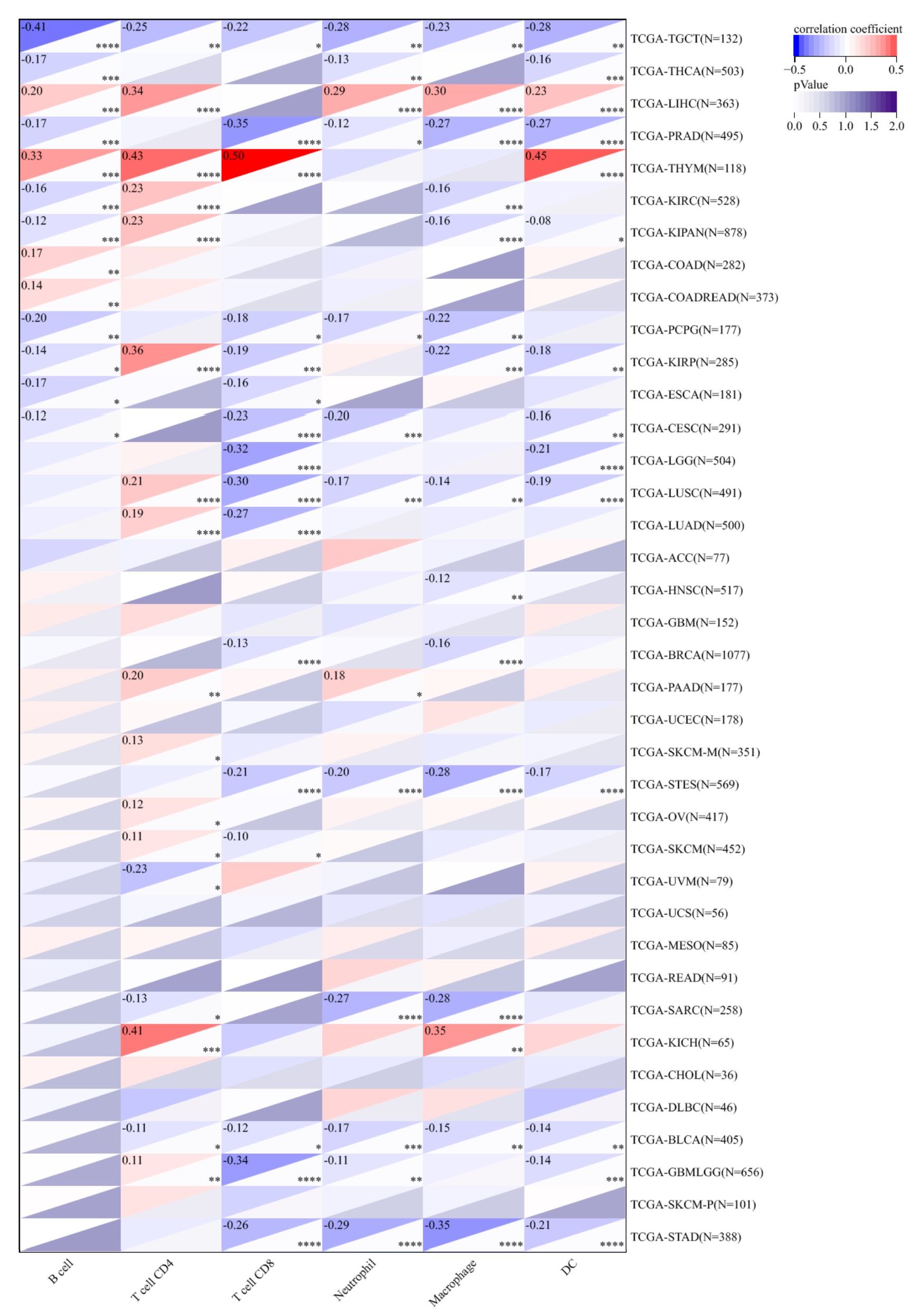

Supplement: Supplementary file 5 [file Image5.tif]

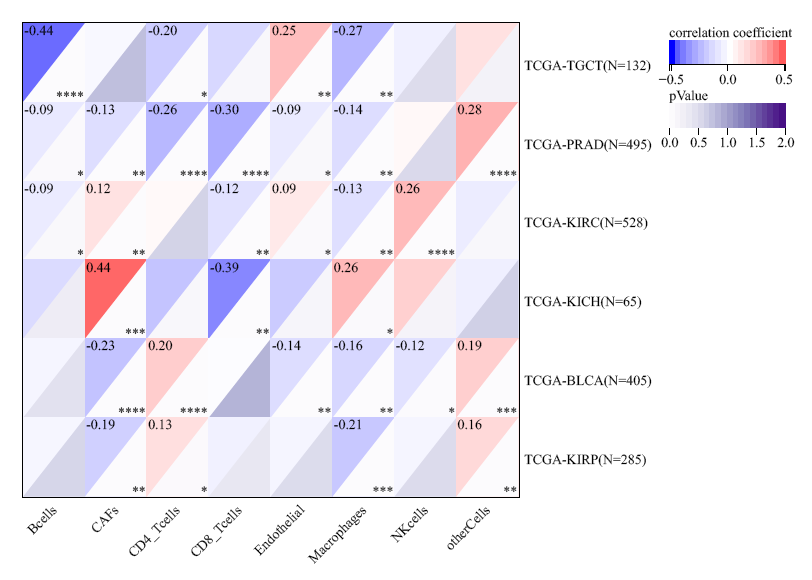

Supplement: Supplementary file 6 [file Image6.tif]

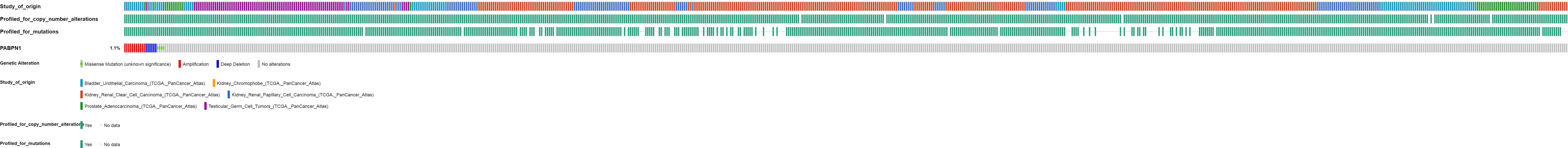

Supplement: Supplementary file 7 [file Image7.png]

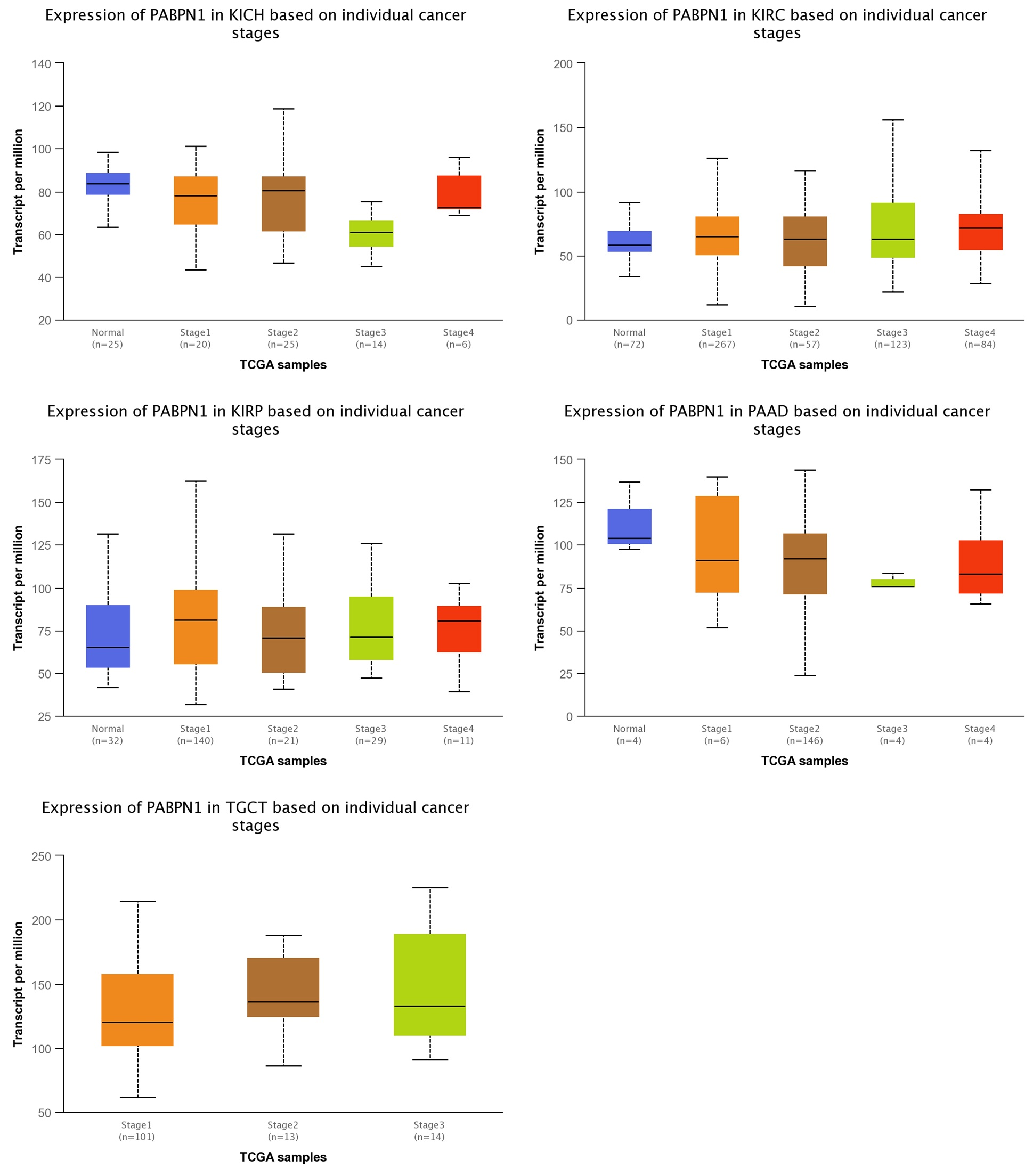

Supplement: Supplementary file 8 [file Image8.tif]

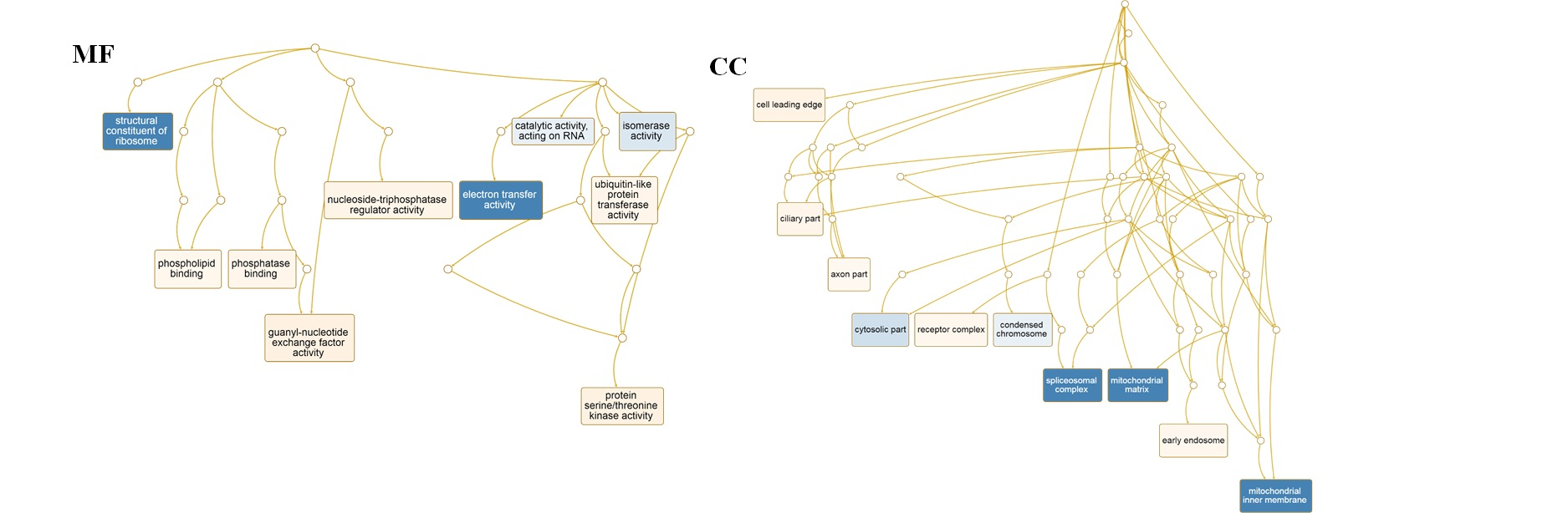

Supplement: Supplementary file 9 [file Image9.tif]

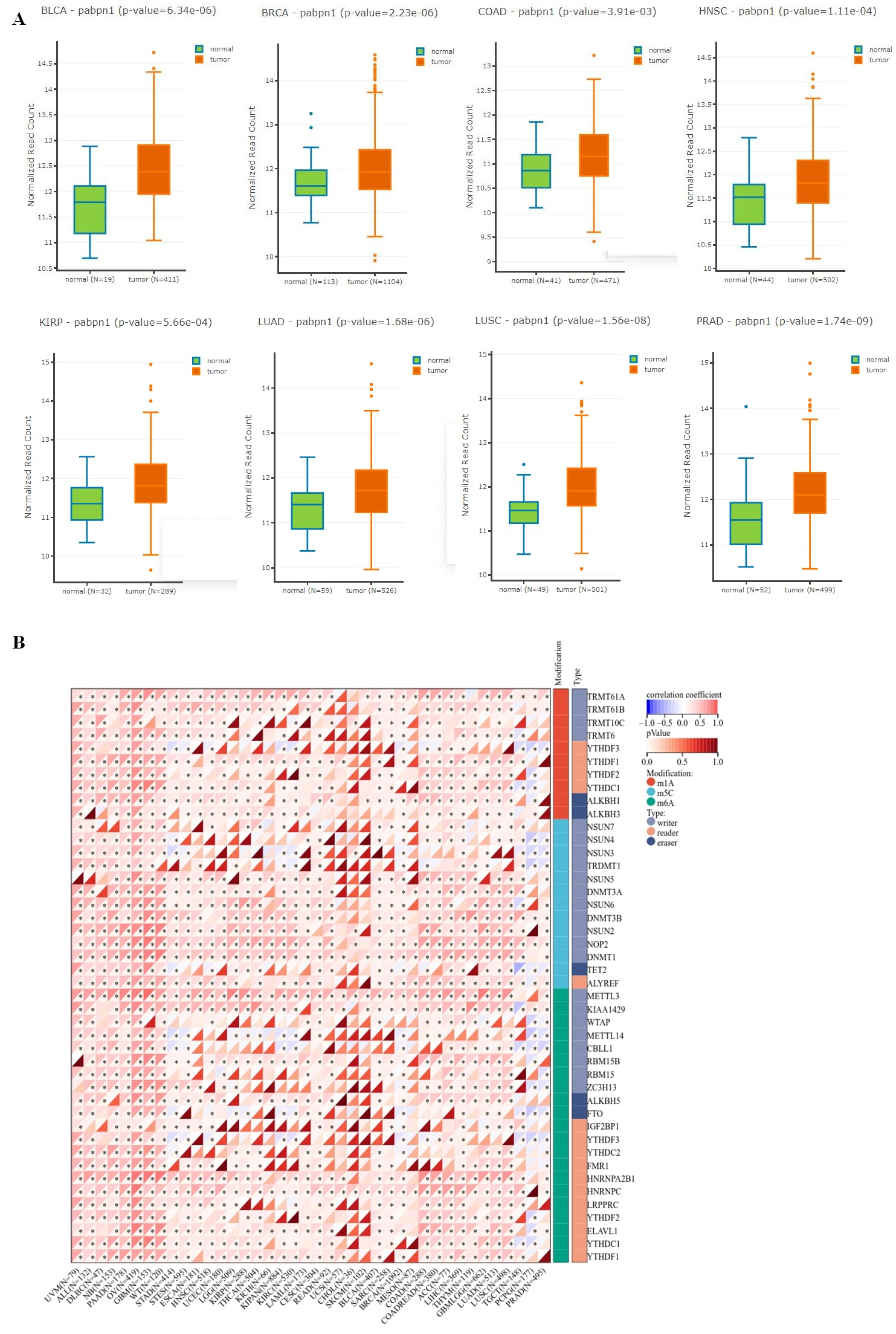

Supplement: Supplementary file 10 [file Image10.tif]

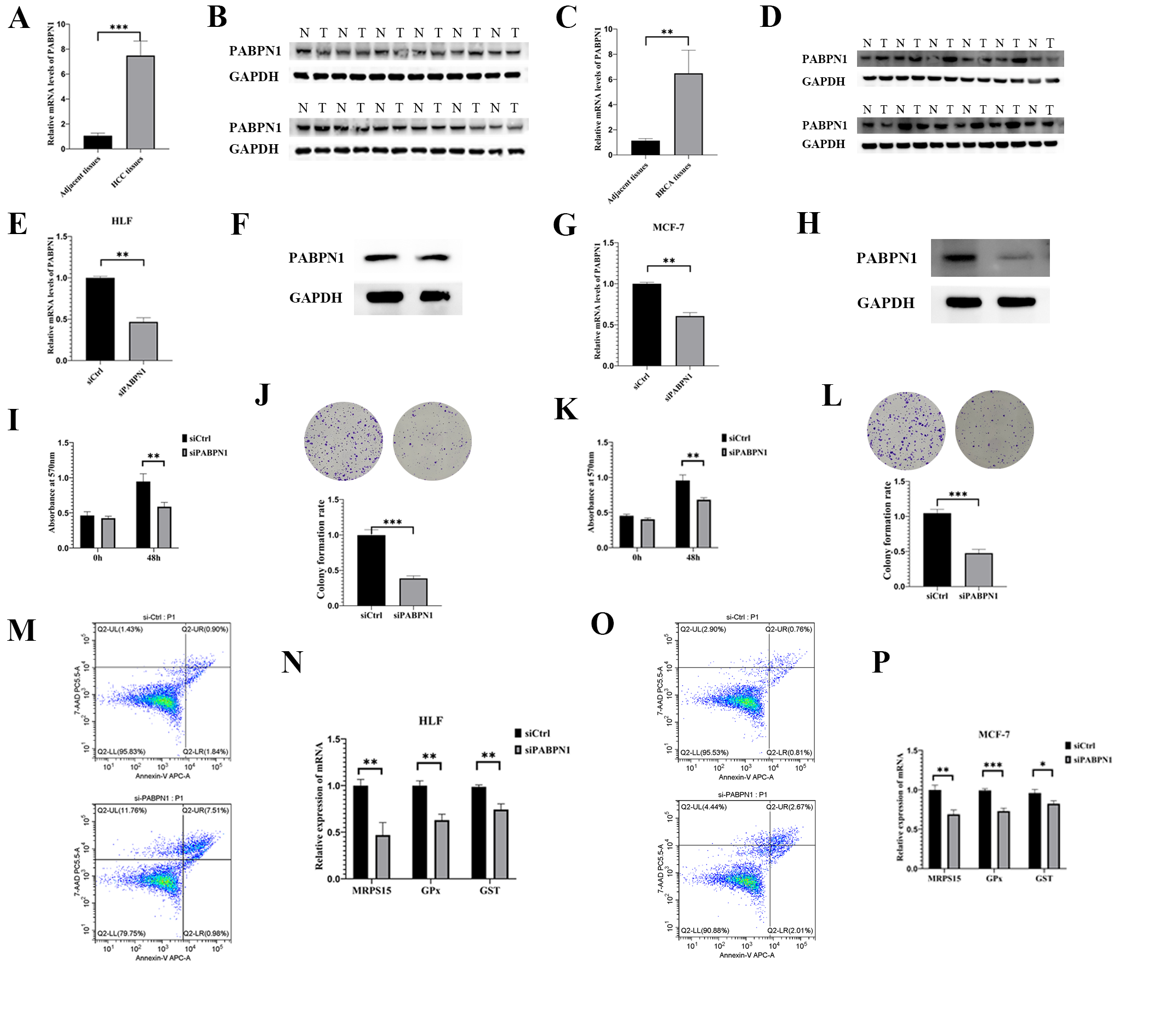

Supplement: Supplementary file 11 [file Image11.tif]
